# Treatment of Diabetes and/or Hypertension Using Medicinal Plants in Cameroon

**DOI:** 10.4172/2167-0412.S2-003

**Published:** 2015-06-08

**Authors:** N Tsabang, CG Yedjou, LWD Tsambang, AT Tchinda, N Donfagsiteli, GA Agbor, PBB Tchounwou, BA Nkongmeneck

**Affiliations:** 1 Center for Research on Medicinal Plants and Traditional Medicine, Institute of Medical Research and Medicinal Plants Studies, Cameroon; 2 Cellomics and Toxicogenomics Research Laboratory, NIH-RCMI Center for Environmental Health, Jackson State University, Jackson, USA; 3 Higher Institute of Medical Technoloy, Nkolonodom, P.O. Box 188 Yaounde, Cameroon; 4 Laboratory of Cellular Signalling, Phytoceuticals, Cancer Prevention and Therapies; College of Science, Engineering and Technology, Jackson State University, Jackson, USA; 5 Millennium Ecological Museum S/C PO. Box. 812 Yaounde, Cameroon

**Keywords:** Medicinal plants, Diabetes, Hypertension, Treatment, Cameroon

## Abstract

Medicinal plants have served as valuable starting materials for drug development in both developing and developed countries. Today, more than 80% of the people living in Africa were depended on medicinal plants based medicines to satisfy their healthcare needs. The main goal of the present study was to collect and document information on herbal remedies traditionally used for the treatment of diabetes and/or hypertension in Cameroon. To reach this objective, data were collected from 328 patients who have been diagnosed at least once by a physician as diabetics and/or hypertension patients. One hundred and eighty two (182) among them took for a period of 10 days different varieties of medicinal plants which were prepared in form of decoction, maceration and infusion and administered orally twice or three times daily.

As result, 70% of patients who used plants were relieved at the end of the treatment. Thirty-three plants have been recorded and documented for the treatment of diabetes and/or hypertension. The results of this study can stimulate a sustainable development by providing the basis for drugs discovery and by documenting biodiversity for long time exploitation.

## Introduction

Hypertension affects approximately 70% of patients with type 2 diabetes, 30% of patients with type 1 diabetes and it is approximately twice as common in persons with diabetes as in non-diabetics [1]. The prevalence of coexistent hypertension and diabetes substantially varies across different socio-cultural and racial groups. The overlap between hypertension and diabetes provokes the risk of vascular complications in the population, and together both pathologies predispose to chronic nephropathy, retinopathy and sexual dysfunction [2]. Diabetes mellitus is an independent risk factor for coronary artery disease, and the risk is markedly increased when hypertension is present. The incidence and prevalence of type 2 diabetes are increasing [3,4]. According to Bild and Teutsch [5], the total number of people with diabetes will rise from 171 million in 2000 to 366 million by 2030. The number of adults with hypertension is predicted to increase by 60% to a total of 1.56 billion people by 2025 [6]. Particularly in Cameroon, epidemiological studies have shown that 10% of the population is diabetics, 90% of them were fat at the beginning of the disease and the prevalence of hypertension adjusted to the age is 16.6% in men and 12.6% in women in urban population. Diabetic nephropathy is the commonest cause of hypertension in patients with type 1 diabetes. Patients with type 2 diabetes can develop renal disease, but hypertension commonly occurs without abnormal renal function and is often associated with central obesity. Insulin resistance and diabetes can precipitate hypertension by stimulating the sympathetic nervous system and the renin–angiotensin system, and promoting sodium retention. Diabetes is also associated with increased proliferation of vascular smooth muscle cells. High blood glucose and elevated blood pressure can impair vascular endothelial cells, leading to increased oxidative stress. Patients with diabetes also have increased vascular reactivity. Ten there is a real need to prevent or to treat quickly both diseases. Herbal remedies are commonly used in Cameroon for the treatment of diseases since they are easily accessible and less expensive compared to conventional drugs. So can plants be required in the management of different types of diabetics and hypertensive patients and what is the prominence of each disease in the population? To answer this question, data were collected from 182 patients, previously diagnosed at least once by a physician as diabetics and/or hypertensive patients who use familiar herbal remedies.

## Methodology

### Patients' criteria selection

Patients admitted in this study have presented a medical book or a bulletin of medical exam that certifies their diabetic and/or hypertensive state at the moment of their selection. During the clinical following up, they have used the familiar blood pressure monitor and/or the familiar glycemic monitor to control daily blood pressure or fasting blood sugar concentration

### Participants

To reach our objective, we selected for this study 182 patients upon 328 who have been diagnosed at least once in medical centers and who took different varieties of herbal remedies and accept the clinical following up for a period of 10 days. They were distributed in three groups: confirmed diabetics, confirmed hypertensive patients and confirmed diabetics with hypertension patients.

Research of minimal efficient therapeutic dosage of each plant used by followed up patients and minimal duration of treatment

Only the more efficient dosage for a given plant prepared with a minimal quantity of plant material for a minimal time of treatment was selected. The daily fasten glycemic and/or the blood pressure of each patient in treatment were measured until the normal fasting glycaemia (0.66 to 1.1 g/l) or the normal blood pressure (110 to 130 mmhg for systolic and 70 to 90 mmhg for diastolic).

Data were collected using an ethnobotanical data form and from 182 upon 328 patients drawn in 28 tribes in Cameroon. All plants recorded here were described in detail with the precision of the quantity of plant material, volume of water, time of ebullition, maceration or infusion, quality of preparation used per dosage, the number uses per day and duration of treatment [7].

## Results

### Clinical following up of diabetes and/or hypertensive patients

One hundred and eighty two (182) confirmed patients (106 diabetics, 66 hypertensive patients and 10 diabetic and hypertensive patients) used familiar herbal remedies. Familiar herbal remedies have regulated the hyperglycemia and/or the high blood pressure for 129/182 patients (70.87%). Oral use of herbal remedies has relieved the hyperglycemia of 80% of patients of NIDD, of 69.44% of patients of IDD on the one hand, the high blood pressure of 46.15% of patients of essential hypertension and 65.07% of patients of secondary hypertension on the other hand. The analysis of the different glycemic values and blood pressure in Table 1 reveal the 2 following tendencies:

A total of 86 abnormal blood pressure values (53 systolic values ≥140 mmHg + 33 diastolic values ≥90 mmHg) upon 152 have been regulated, representing 56,57%. However 22 of such values have persisted after the treatment, representing 14.47%. At last 33 diastolic normal values (21.71%) remained constant until the end of the treatment. At last a normal systolic value that represents 0.65% remained constant until the end of the treatment. For the two diseases 64.17% of glycemic values and blood pressure values have been regulated.

These increased percentages constitute an element that sustains the use of herbal remedies in the treatment of the two pathologies. However because of the disparity observed in the results of treatment, it is necessary to precise that the use of plants must be done under a control of a physician. Stop the treatment, after a time to determine from that the glycaemia and/or the blood pressure become normal. This precision must avoid the excessive use of herbal remedies and the danger of hypoglycemia and hypotension.

### Plants used by the 182 followed up patients

Thirty-three plants were used by the 182 followed up patients. The minimal efficient dosage of each of these plants was taken in consideration. It is expressed sometimes in function of patients' weight and is subsequently described in details in Table 2. This table shows the most useful plants in term of the number of patients and/or diseases relieved.

## Discussion

The abandon of several foods in benefit of sweet coffee and milk by the urban populations including hypoglycemic garden eggs *Solanum melongena* in the form of a decoction called ‘medip-me-zon’ in Boulou tribe, the soup with garden eggs of fufu called ‘ashu’ and ‘abalah ‘respectively in North West and West regions and the soup of fufu corn with hypoglycemic *Corchorus olitorius* called ‘lalo’ in Fulfulbe and Kenekene in Eton and the lack of physical exercises can explain the increase prevalence of the diabetes and hypertension in towns.

The high percentage of regulated or relieved patients (70, 87%) depends on many factors including the aggravation of the diseases by the development of complications, the causes of diseases and the mechanisms of the herbal remedies including insulin-secretion and peripheral action. Most of the unrelieved patients have developed the disease for a long time.

### Insulin-secretion

**Figure 1:**
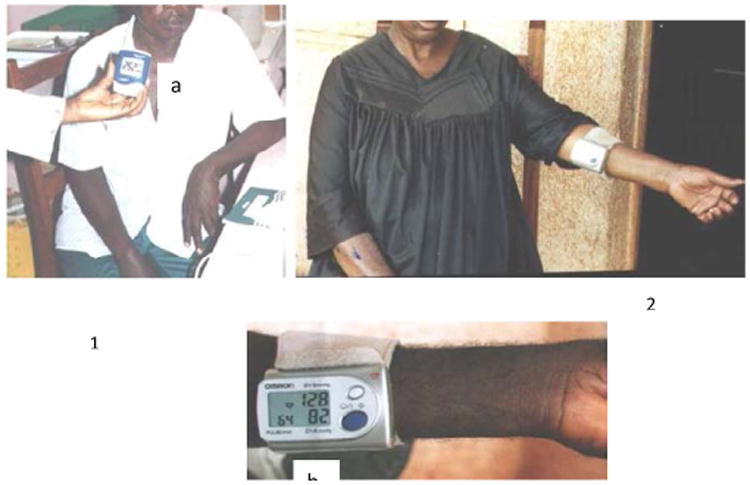
Diabetic; photograph 2 Hypertensive patient: (a) Glycemic monitor; (b) Blood pressure monitor.

It is the pancreatic action that is possible if β- cells still available (Figure 1). In the Table 2 the plants* endowed of this mechanism of action can treat only the type 2 diabetes. These plants are comparable to oral hypoglycemic products and produce normal diabetic insulin [7]. But their prolonged use provokes the death of β-cells due to functional overwork. Progressively the patient passes from type 2 (NIDD) to type 1 (IDD).

### Peripheral effect

**Figure 2:**
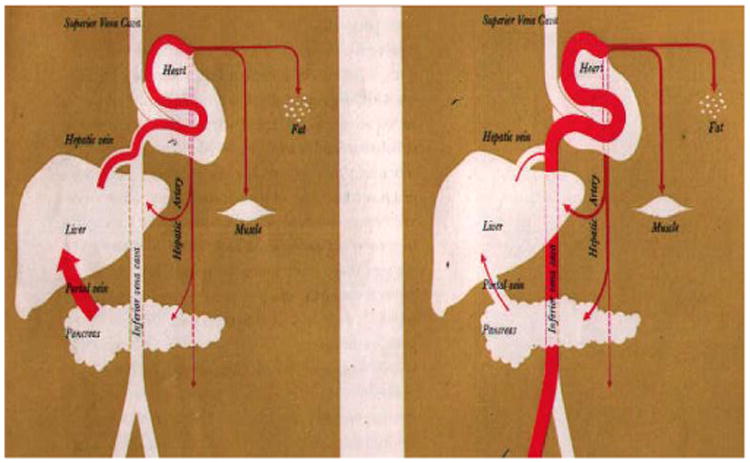
Mechanisms' comparison action of normal diabetic insulin (left) and injectable insulin (right) (Upjohn).

It is the extrapancreatic action (as injected insulin) (Figure 2). The Table 2 present the plants with this action.

### Insulin-secretion and peripheral effect

The two mechanisms present by the plants* of Table 2.

### Essential hypertension

*Phyllanthus niruri* is an inhibitor of conversion enzyme (ICE). These compounds are hypotensive and they interfere with the liberation of angiotensin. The Heart Outcomes Prevention Evaluation study showed that inhibition of angiotensin-converting enzyme in patients with type 2 diabetes reduces the risk of vascular complications [8]. Therefore this plant can be required in the management of these patients, but lifestyle modification and weight management are key components to reduce glycemia and control blood pressure.

Amaechina and Omogbai [9] reported that intravenous administration of the aqueous extract of the leaves of *Phyllanthus amarus* (5-80 mg/kg) to anesthetized male rabbits produced a significant fall in mean diastolic, systolic, and mean arterial pressures in a graded dose-response manner. The dose of 5 mg/kg produced the least hypotensive effect, causing a fall in mean diastolic, systolic, and mean arterial pressure of 13.3 ± 3.1, 19.7 ± 5.4, and 14.3 ± 3.4 mmHg, respectively, whereas the dose of 80 mg/kg produced the greatest fall in mean diastolic, systolic, and mean arterial pressure of 49.7 ± 7.9, 45.5 ± 9.5, and 48.00 ± 6.5 mmHg, respectively. The extract produced greater depressant effect on the diastolic blood pressure than the systolic blood pressure.

### Secondary hypertension

Some plants 1 in Table 2 low the rate of cholesterol and/or blood triglycerides, responsible of certain secondary or certain hypotensive properties which provoke peripheral vasodilatation hypertensions contain. Therefore they can treat hypertensions due to arteriosclerosis. *Rauvolfia vomitoria* reacts like a central antihypertensive product; as vasodilator of coronary vessels and as a regulator of cardiac rhythm in patients suffering of tachycardia. *Allium cepa* is a diuretic; it treats some hypertensive patients by facilitating the blood circulation and prevents thrombosis, responsible of some secondary hypertensions. This favorable effect can be partially explained by the physio-pathological mechanisms conceivable in African population like hypervolemia (strong blood concentration in salt), the sensibility in salt, the excessive consumption of salt and the climate conditions. In anesthetized rats, the crude extract of the leaves of *Moringa oleifera* caused a fall in systolic, diastolic, and mean blood pressure in a dose-dependent manner. It was also established that thiocarbamate and isothiocyanate fractions of this crude extract were responsible for the antihypertensive activity. Sacks, et al. and Mojiminiyi et al. [10,11] reported the antihypertensive effect of calyx of *Hibiscus sabdariffa*. The antihypertensive effects of the crude extract have been attributed to mediation through acetylcholine and histamine like dependent mechanism through direct vasorelaxant effects [12]. Earlier report showed that the petal crude extract had a direct relaxant effect on the aortic smooth muscle of rats [13]. The chronic administration of aqueous extract reports to reverse cardiac hypertrophy in renovascular hypertensive rats [14].

Clinical trials of the plant extract in human being show trustworthy evidence of antihypertensive effects. A standardized doses of 9.6 mg per day given to 39 patients, 50 mg per day given to the same number of patients don't show significant difference relative to hypotensive effects, antihypertensive effectiveness and tolerability [15].

## Conclusion

In Cameroon, the persisted economic crisis, the insulin-resistance, the resistance of some hypertensions to antihypertensive products and certain germs to synthetic antibiotics has reinforced the recourse to traditional medicine in both urban and rural populations.

The clinical follow up of diabetics and/or hypertensive patients in treatment with familiar herbal remedies led to the identification of 33 hypoglycemic and/or antihypertensive plants. In general the oral use of these 33 plants have reduced the hyperglycemia of 80% of NID patients, 69.4% of IDD patients on the one hand; the high blood pressure of 46.1% of patients of essential hypertension and 65.1% of patients of secondary hypertension on the other hand. For the two diseases 172 glycemic and tension values upon 268 were regulated representing 64.17% [16]. Some plants including *Momordica charantia*, *Sclerocarya birrea* and *Ceiba pentandra* have revealed two interesting mechanisms of action: pancreatic and extra-pancreatic in the treatment of diabetes. *Phyllanthus niruri* is an inhibitor action for the production aldosterone responsible of certain severe hypertensions [17].

The knowledge of bioactive plants can contribute to the better treatment of patients and the improvement of the quality of socio-sanitary allowance administrated to them [7]. But many other plants need to be investigated. It is also necessary to carry out advanced investigations on the study of different toxicities of the herbal remedies.

## Figures and Tables

**Table 1:** Distribution of glycemic and blood pressure values of the followed up patients according to sex.

	SEXES		Glycemic values					Blood pressure values						
			NIDD		IDD		T	SYSTOLIC			DIASTOLIC			T
			RV	NRV	RV	NRV		NV	RV	NRV	NV	RV	NRV	
**GV and TV/S**	**M**	91	28	12	15	3	**58**	1	25	9	15	17	5	**72**
	**W**	91	30	10	13	5	**58**		28	13	18	16	5	**80**
**Total**		182	58	22	28	8	**116**	1	53	22	33	33	10	**152**

M: Men; W: Women; NIDD: Non insulino-dependent diabetes; IDD: Insulino-dependent diabetes; RV: Regulated values; NRV: Not regulated values; NV: Normal values. T: Total; GV or BPV/S: Glycemic values and blood pressure values per sex.

**Table 2:** Distribution of the followed up patients according to the plants used and their healer condition.

Scientific names	Patients in treatment by familiar medications									
	NIDD		IDD		SHT		EHT		D-H	
	RP	URP	RP	URP	RP	URP	RP	URP	RP	URP
1. *Phyllanthus niruri* x^1^							4	3	3	
2. *Phyllanthus amarus*^1^									1	
3. *Mucuna pruriens* x*	2				4	1			1	
4. Rhizophora racemosa*	2									
5. *Anacardium occidentale* x 1	5	2	3	1						
*Persea americana*	3									
*Pterocarpus osun*	4	1								
6. *Momordica charantia* x●▫	2	1	4							
7. *Asystasia gangetica*					2	3				
8. *Rauvolfa vomitoria* x					6	3				
9. *Laportea ovalifolia*			4	1						
10. *Morinda lucida* x					3	1			2	3
11. *Aloe barteri* x	7	1			3					
12. *Ceiba pentandra* ●▫	3	1								
13. *Moringa oleiracea*							2	1		
14. *Allium cepa* x*	5	1			3					
15. *Spathodea campanulata*●	3	3								
16. *Hallea stipulosa*					2	1				
17. *Hallea inermis*					4	4				
18. *Pterocarpus soyauxii*	3									
19. *Brassica oleracea* (Brassicaceae) associated to 20. *Citrus grandis* (Rutaceae)	3									
*23Allium sativum*^1^ (Liliaceae)					2	2				
21. *Vernonia glabra* (Asteraceae)	2				2	2				
*26. Aloe buettneri* (Liliaceae)	2	1			2	1				
*27. Catharanthus roseus* x*	5									
28. Azadirachta indica*	3									
*29. Sclerocarya birrea* x* ●▫			6	5						
*30. Hibiscus sabdariffa (family)*			3	2						
*31. Voacanga africana* (Apocynaceae)					1	4				
*32. Corchorus olitorius (family)*	3	2								
*33. Solanum melongena* x 1 *(family)associated with Spathodea campanulata (family)*			5	2						
**Total**	**57**	**13**	**25**	**11**	**34**	**22**	**6**	**4**	**7**	**3**

* Plants with pancreatic effect;

● plants with extra-pancreatic effect;

▫ plants with the two effects;

1 plants which low cholesterol and/or blood triglycerides responsible of secondary hypertension; x plants which relieve at least five patients

NIDD: Non Insulin-Dependent Diabetes; IDD: Insulin-Dependent Diabetes; SHT: Secondary Hypertension; EHT: Essential Hypertension; D-H: Diabetics with Hypertensive Patients; RP: Relieved Patients; URP: Unrelieved Patients
